# Distribution and Accessibility of Hospital‐Based Emergency Departments for Paediatric Patients in Rural and Remote Western Australia: A Geographic Analysis

**DOI:** 10.1111/1742-6723.70302

**Published:** 2026-06-14

**Authors:** Hanin Bakhaider, Somayyeh Azimi, Marc Tennant, Estie Kruger

**Affiliations:** ^1^ Paediatric Dentistry Department, Faculty of Dentistry King Abdulaziz University Jeddah Saudi Arabia; ^2^ International Research Collaborative ‐Health and Equity, School of Health and Clinical Sciences University of Western Australia Perth Western Australia Australia

**Keywords:** geographic information system, paediatric emergency care, rural and remote health, spatial accessibility, Western Australia

## Abstract

**Objective:**

This study examined the distribution of emergency departments relative to remoteness and socioeconomic disadvantage and explored service availability for children in non‐metropolitan Western Australia.

**Methods:**

We conducted a spatial analysis using the Australian Government datasets. Child population counts and emergency facility locations were integrated into Quantum Geographic Information System and classified by remoteness using the Modified Monash Model (MMM) and by area‐level socioeconomic disadvantage using the Index of Relative Socioeconomic Disadvantage (IRSD). Buffer zones were generated around each facility to quantify the proportion of children residing within specified distance thresholds. Service availability was assessed using facility‐to‐child and bed‐to‐child ratios.

**Results:**

Emergency department availability declined with increasing remoteness: MMM5 contained the largest share of facilities (42.6%), followed by MMM6 (31.2%) and MMM7 (26.2%). However, ratios appeared more favourable in very remote areas, with facility‐to‐child ratios improving from 1:848 (MMM5) to 1:741 (MMM7). Bed availability showed a similar pattern, ranging from 1:83 in MMM5 to 1:60 in MMM7. Socioeconomic analyses revealed a concentration of facilities and bed capacity in the most disadvantaged areas, with IRSD 1 hosting 57.4% of all facilities and 35.8% of total bed capacity.

**Conclusion:**

Geographic and socioeconomic inequities in emergency care provision persist across rural and remote Western Australia. Apparent improvements in ratios in very remote areas are driven by low population density rather than greater service investment. These findings support a shift to needs‐based paediatric emergency service planning, using remoteness and IRSD‐stratified proximity and capacity measures to prioritise communities with the greatest access shortfalls.

## 1 Introduction

One in four Australian children aged 0–14 years resides outside major cities [1], representing a substantial and consistently underserved population. Australia's vast landmass and low population density outside metropolitan areas present significant challenges for the delivery of health services to rural and remote (RR) communities. This is closely tied to the uneven distribution of medical professionals, with RR areas experiencing shortages across medical disciplines, resulting in significantly lower utilisation of health services amongst rural residents relative to urban Australians [2].

Emergency departments (EDs) are the ‘front door’ to the Australian health system, providing high‐quality immediate care for acute illness and injury [3]. Their role is increasingly important as demand continues to rise. National data show steady growth in emergency presentations over the past 5 years, increasing from 8.2 million in 2019–2020 to 9.0 million in 2023–2024, representing an average annual growth rate of 2.3% [4]. Paediatric demand has also grown, with public hospital ED presentations amongst children aged ≤ 14 years increasing from 1.69 million in 2017–2018 to 1.79 million in 2023–2024 [4, 5]. Rural emergency services are provided through a range of facilities, including hospitals, general practises, nurse‐led clinics, pre‐hospital/retrieval services and stand‐alone urgent care centres [6], with substantial variation in capability. However, when patient needs exceed local capacity, transfer to other facilities within the emergency care network is required for specialised management [7].

Similarly, ED presentations are not uniformly distributed across the Australian population, with significant disparities observed across key demographic, geographic and socioeconomic factors. Utilisation rates are particularly high amongst paediatric patients, with children aged 4 and under accounting for the second highest presentation rates nationally [8]. Furthermore, children living in remote Australia are more than twice as likely to present to an ED as their counterparts in major cities [8]. Likewise, individuals residing in the most socioeconomically disadvantaged areas have double the rate of emergency presentation [4]. This substantial demand for emergency care disproportionately impacts the most remote and socioeconomically disadvantaged populations, highlighting the need for research on equitable service distribution in these areas.

Western Australia (WA) provides a unique opportunity to investigate the impact of remoteness and area‐level socioeconomic status on timely access to emergency care. The state's vast geography is four times the size of Texas, USA, and its sparse population distribution averages one person per five square kilometres. Approximately 80% of its 2.6 million residents live in the capital city, Perth, whilst the remaining 20% are dispersed across 2.5 million square kilometres. The extreme rurality of WA's population distribution presents significant challenges for the timely delivery of emergency care [9].

The specific focus on children is essential, given the substantial burden of emergency care utilisation amongst them [10]. As children grow and develop, their evolving motor skills and behaviours expose them to a wider range of injury mechanisms, which can produce profound and lifelong consequences [11, 12, 13, 14]. Given the importance of the first 1000 days of life [15], equitable access to healthcare for children is essential. However, Australian evidence has largely focused on service activity (e.g., ED presentations) or broad regional summaries [4], and few studies have assessed remoteness and socioeconomic disadvantage at a fine spatial resolution [16]. Accordingly, this study integrates spatial datasets with child population, socioeconomic and remoteness measures to quantify geographic and socio‐economic inequalities in paediatric access to ED care.

## 2 Methodology

The study data were collected from Australian government open‐source databases. An exemption was obtained from the University of Western Australia Human Research Ethics Committee (2025/ET000116).

*Population and ED data:* De‐identified SA1‐level counts of children aged 0–14 years, consistent with the ABS child population reporting categories [17], were drawn from the 2021 ABS Census [18]. RR public hospitals offering emergency services were identified from the WA Department of Health website in September 2025 [19], and ED coordinates were obtained from Google Maps. Analysis was restricted to hospital‐based EDs listed on the WA Department of Health website as providing 24‐h emergency services at the time of data extraction. The total number of hospital beds was used as a proxy for ED capacity, as ED‐specific bed data were not consistently reported. However, service capability varies as described by the Australasian College for Emergency Medicine (ACEM) Role Delineation Framework [7] and the WA Country Health Service Hospital Service Matrix, which classifies sites by service level and reports paediatric ED role delineation [20] (Table S1).

*Remoteness classification*: Remoteness was assessed using the Modified Monash Model (MMM), which classifies areas into seven categories from MM1 (major cities) to MM7 (very remote areas). It is based on the Australian Statistical Geography Standard‐Remoteness Areas (ASGS‐RA) framework [21]. The MMM was selected over alternative remoteness indices due to its direct relevance to health service planning and resource allocation [21]. To focus on RR access disparities, this study aggregated the paediatric population and EDs across MM5–MM7.

*Sociodemographic characteristics:* Area‐level socioeconomic status was classified using the Socioeconomic Index for Areas (SEIFA), specifically the Index of Relative Socioeconomic Disadvantage (IRSD), which summarises population‐level indicators of disadvantage within each geographic area. This index ranks areas into 10 deciles. IRSD 1 represents areas with the greatest relative socioeconomic disadvantage, characterised by lower income, lower educational attainment, higher unemployment and a greater proportion of low‐skilled occupations, whereas IRSD 10 represents areas with the least relative socioeconomic disadvantage [22]. For analysis, IRSD deciles were grouped into five ordinal groups, with group 1 representing the most disadvantaged areas and group 5 representing the least disadvantaged areas.

*Geographic and statistical analysis:* The WA landmass was mapped using the Australian Statistical Geography Standard (ASGS) Edition 3 and SA1 digital boundary shapefiles [23]. SA1s are the smallest ABS census unit and involve an average population of 400 people; however, in RR areas SA1s have smaller populations [24]. Spatial processing was undertaken in Quantum Geographic Information System (QGIS) (version 3.40, Bratislava). SA1 polygons were joined with MMM, IRSD and SA1‐level paediatric population data. The location of each ED was added to the resultant map to enable comprehensive analysis at the SA1 level.

For distance‐based analyses, SA1 centroids and Euclidean buffers were used. Euclidean distance is commonly applied in healthcare accessibility studies and correlates well with network‐based distance [25]. Given WA's large geographic scale and sparse population distribution, a single fixed catchment does not adequately reflect access across RR settings [26]. We therefore applied graduated 25 km, 50 km and 100 km buffers to represent local, extended and longer‐distance access thresholds. These distances are consistent with prior Australian research and with WA policy‐relevant concepts of substantial travel, including the > 100 km threshold used in Patient Assisted Travel Scheme guidance [27]. A 25 km catchment has been used in Australian emergency access studies to represent local proximity to services [16], whilst 50 km and 100 km buffers have been used in RR WA to assess health service access, reflecting variation in travel tolerance and the need to represent extended journeys in sparsely populated areas [28, 29].

To estimate the proportion of children residing within 25, 50 and 100 km of an ED in the absence of address‐level data, we applied a probabilistic sampling approach using QGIS's ‘random points in polygons’ procedure. For each SA1, we generated a set of simulated child residential locations within the SA1 boundary, with the number of points scaled to the SA1 child population [25]. We then overlaid these points with the ED buffer zones and calculated coverage as the proportion of children captured within each buffer. This approach reduced bias from assigning all residents in large, sparsely populated SA1s to a single centroid [30].

All data were exported from QGIS for further analysis in Microsoft Excel (Version 16.101.1). The ED and bed‐to‐population ratios are standard measures for expressing service supply within a specific area. These provide straightforward indicators that are easy to interpret by policymakers.

## 3 Results

The 879 SA1s formed non‐overlapping regions across WA, with a total child population of 53,082 distributed across small rural towns (22,057), remote communities (19,163) and very remote communities (11,862). A total of 61 hospital‐based EDs were geolocated (Table 1).

**TABLE 1:** Distribution of emergency departments and bed capacity across Modified Monash Model (MMM) categories and Index of Relative Socio‐economic Disadvantage groups, WA.

Modified Monash Model (MMM)	Number of emergency departments *n* (%)	Bed numbers *n* (%)^‡^
Small rural town (MMM5)	26 (42.6%)	265 (35.1%)
Remote communities (MMM6)	19 (31.1%)	292 (38.7%)
Very remote communities (MMM7)	16 (26.2%)	197 (26.1%)
Index of Relative Socioeconomic Disadvantage (IRSD)^†^		
1	35 (57.3%)	270 (35.8%)
2	12 (19.7%)	237 (31.4%)
3	8 (13.1%)	154 (20.4%)
4	3 (4.9%)	26 (3.4%)
5	2 (3.3%)	60 (7.9%)

^†^ 1.6% ED and 0.9% bed capacity were missing IRSD classification.

^‡^ Bed numbers represent total hospital inpatient beds.

### 3.1 Geographic Distribution

**FIGURE 1:**
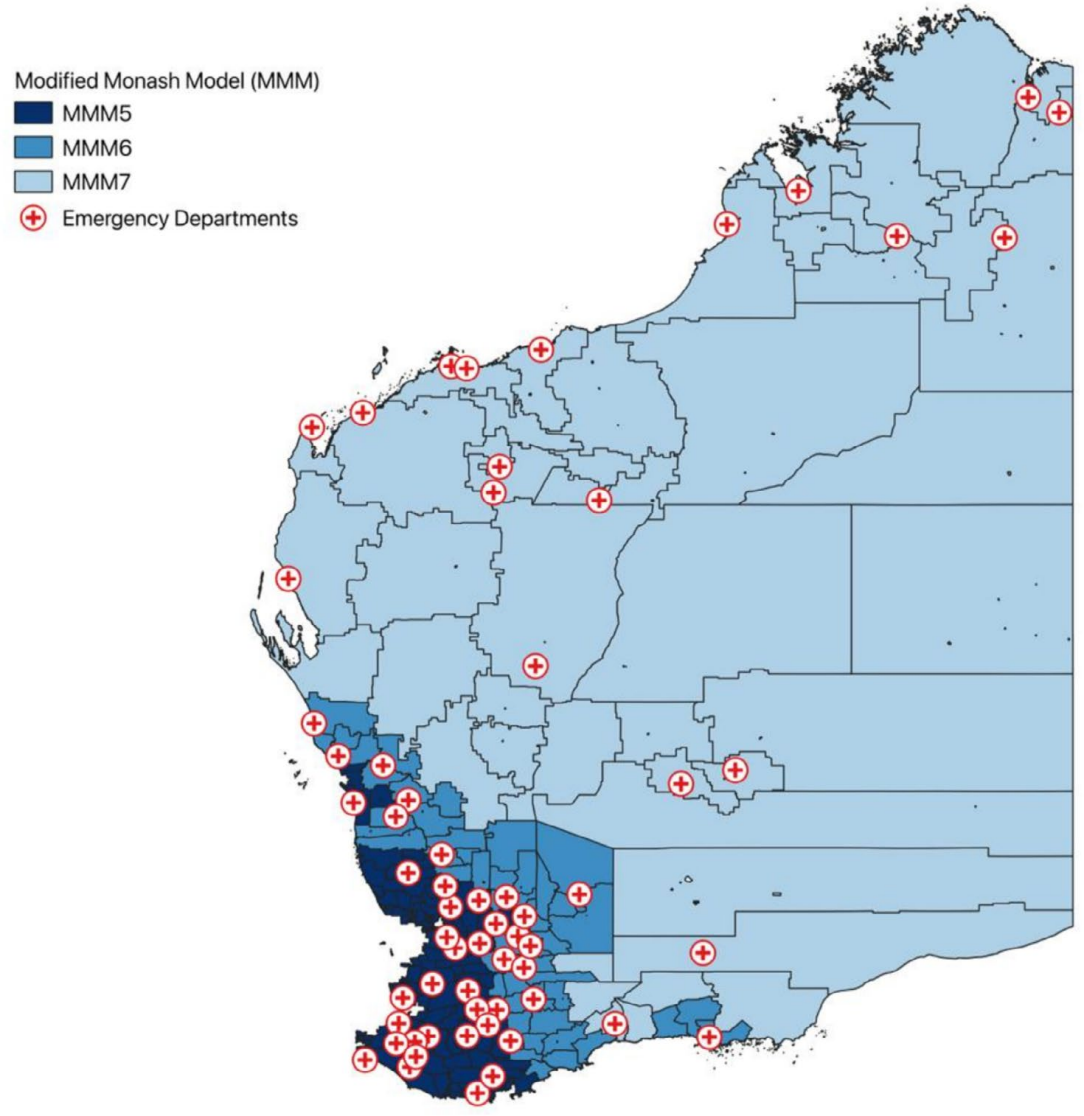
Distribution of emergency departments by remoteness categories.

Across the study area, MMM5 contained the largest share of EDs (42.6%; *n* = 26), followed by MMM6 (31.1%; *n* = 19) and MMM7 (26.2%; *n* = 16) (Figure 1). A similar declining pattern was observed in the distribution of the child population, with MMM5 accommodating 41.5% of all children (*n* = 22,057), MMM6 accounting for 36.1% (*n* = 19,163) and MMM7 comprising 22.3% (*n* = 11,862). Patterns in bed capacity showed a different gradient across remoteness categories. Although MMM5 hosted the highest number of EDs, it accounted for a smaller share of total hospital beds (35.1%; *n* = 265). In contrast, MMM6, despite having fewer EDs than MMM5, contained the largest proportion of bed capacity (38.7%; *n* = 292). MMM7 contributed 26.1% (*n* = 197) of all hospital beds. The analysis of service provision within the defined study boundaries reveals a nuanced pattern of resource allocation. The ED‐to‐child ratio was lowest in MMM6, with one facility per 1009 children, compared with 1:848 in MMM5 and 1:741 in MMM7. These ratios are largely driven by very small population denominators and do not indicate greater functional service availability. When service capacity was standardised by bed availability, a similar denominator effect was observed in the bed‐to‐child ratios and a direct gradient emerged with remoteness. MMM5 had one bed for every 83 children, whereas MMM6 demonstrated a higher level of bed availability with one bed per 66 children. The very remote zone, MMM7, exhibited the most favourable per‐capita bed capacity.

### 3.2 Socio‐Economic Distribution

**FIGURE 2:**
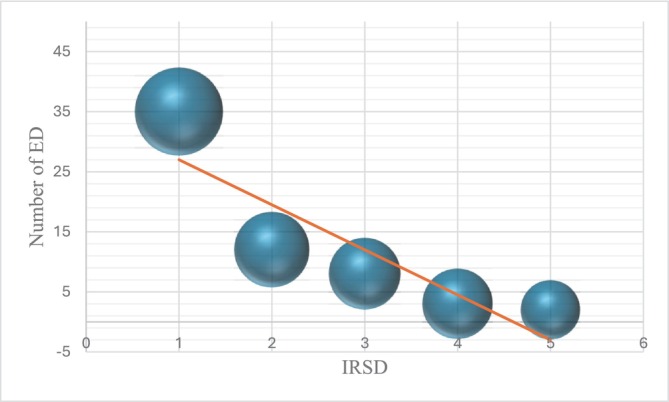
Emergency‐department availability across IRSD decile: Bubble plot with fitted linear trend. Count of ED (*y*‐axis), IRSD decile (1 = most disadvantaged, 5 = least) (*x*‐axis), with bubble size scale to population within each IRSD decile.

Table 1 and Table 2 show the distribution of EDs and children in relation to area‐level socioeconomic status. A substantial proportion of children residing in RR areas fell within IRSD 1, indicating that many of the most geographically isolated communities are also amongst the most socioeconomically disadvantaged. Importantly, emergency‐care resources were strongly concentrated in socioeconomically disadvantaged areas. IRSD 1 contained 57.3% of all EDs. IRSD 2 contributed a further 19.7% of EDs. Resource availability declined steadily with increasing socioeconomic advantage: IRSD 3 had 13.1% of EDs, whilst IRSD 4 and IRSD 5 had only 4.9% and 3.3% of EDs, respectively. This pattern was mirrored in per‐capita measures, with ED‐to‐population ratios worsening from 1:433 in IRSD 1 to 1:3420 in IRSD 5 and bed‐to‐population ratios declining from 1:56 to 1:114 across the same gradient. A small number of SA1s had missing IRSD assignments (0.7% of children) and contained one facility (1.6%). (Figure 2).

**TABLE 2:** Number and percentage of children within 25‐, 50‐ and 100‐km of an emergency department, stratified by MMM and IRSD; ED‐to‐population ratios, bed capacity‐to‐population ratios and ED per 1000 population.

	Sum of population *n* (%)^‡^	ED:population ratio	Bed capacity: population ratio	ED per 1000 population	Bed capacity per 1000 population^†^
Across Western Australia SA1 (*n* = 879)					
MMM: MMM5	22,057 (41.5%)	1:848	1:83	1.18	12.01
MMM6	19,163 (36.1%)	1:1009	1:66	0.99	15.24
MMM7	11,862 (22.3%)	1:741	1:60	1.35	16.61
IRSD: 1	15,143 (28.5%)	1:433	1:56	2.31	17.83
2	11,073 (20.9%)	1:923	1:47	1.08	21.40
3	9973 (18.8%)	1:1247	1:65	0.80	15.44
4	9651 (18.2%)	1:3217	1:371	0.31	2.69
5	6840 (12.9%)	1:3420	1:114	0.29	8.77
Total	53,082	1:870	1:70	1.15	14.20
25 km buffer SA1 (*n* = 545)					
MMM5	12,276 (33.5%)	1:472	1:46	2.12	21.59
MMM6	17,031 (46.5%)	1:946	1:60	1.06	16.73
MMM7	7349 (20.1%)	1:459	1:37	2.18	26.81
IRSD: 1	10,084 (27.5%)	1:288	1:37	3.47	26.78
2	7561 (20.6%)	1:630	1:32	1.59	31.35
3	6916 (18.9%)	1:865	1:45	1.16	22.27
4	6465 (17.6%)	1:2155	1:249	0.46	4.02
5	5563 (15.2%)	1:2782	1:93	0.36	10.79
Total	36,656 (69.1%)	1:611	1:49	1.64	20.38
50 km Buffer SA1 (*n* = 670)					
MMM5	17,489 (40.3%)	1:673	1:66	1.49	15.15
MMM6	18,097 (41.7%)	1:1005	1:63	0.99	15.75
MMM7	7799 (17.9%)	1:487	1:40	2.05	25.26
IRSD: 1	11,602 (26.7%)	1331	1:43	3.02	23.27
2	9011 (20.8%)	1:751	1:38	1.33	26.30
3	8472 (19.5%)	1:1059	1:55	0.94	18.18
4	8014 (18.5%)	1:2671	1:308	0.37	3.24
5	6188 (14.3%)	1:3094	1:103	0.23	9.70
Total	43,385 (81.7%)	1:723	1:58	1.38	17.22
100 km buffer SA1 (*n* = 779)					
MMM5	21,158 (43.2%)	1:814	1:80	1.23	12.52
MMM6	18,907 (38.6%)	1:995	1:65	1.00	15.44
MMM7	8895 (18.2%)	1:556	1:45	1.80	22.15
IRSD: 1	12,635 (25.8%)	1:361	1:47	2.77	21.37
2	10,423 (21.3%)	1:869	1:44	1.15	22.74
3	9610 (19.6%)	1:1201	1:62	0.83	16.02
4	9362 (19.1%)	1:3121	1:360	0.32	2.78
5	6753 (13.8%)	1:3377	1:113	0.30	8.88
Total^‡^	48,960 (92.2%)	1:803	1:65	1.25	15.40

Abbreviations: IRSD; Index of Relative Socio‐economic Disadvantage, MMM; Modified Monash Model, SA1; Statistical Area level 1.

^†^ Bed capacity represents total hospital inpatient beds.

^‡^ IRSD classifications were missing for 0.7% of the target population.

### 3.3 Geographic Accessibility Across Distance Buffers

**FIGURE 3:**
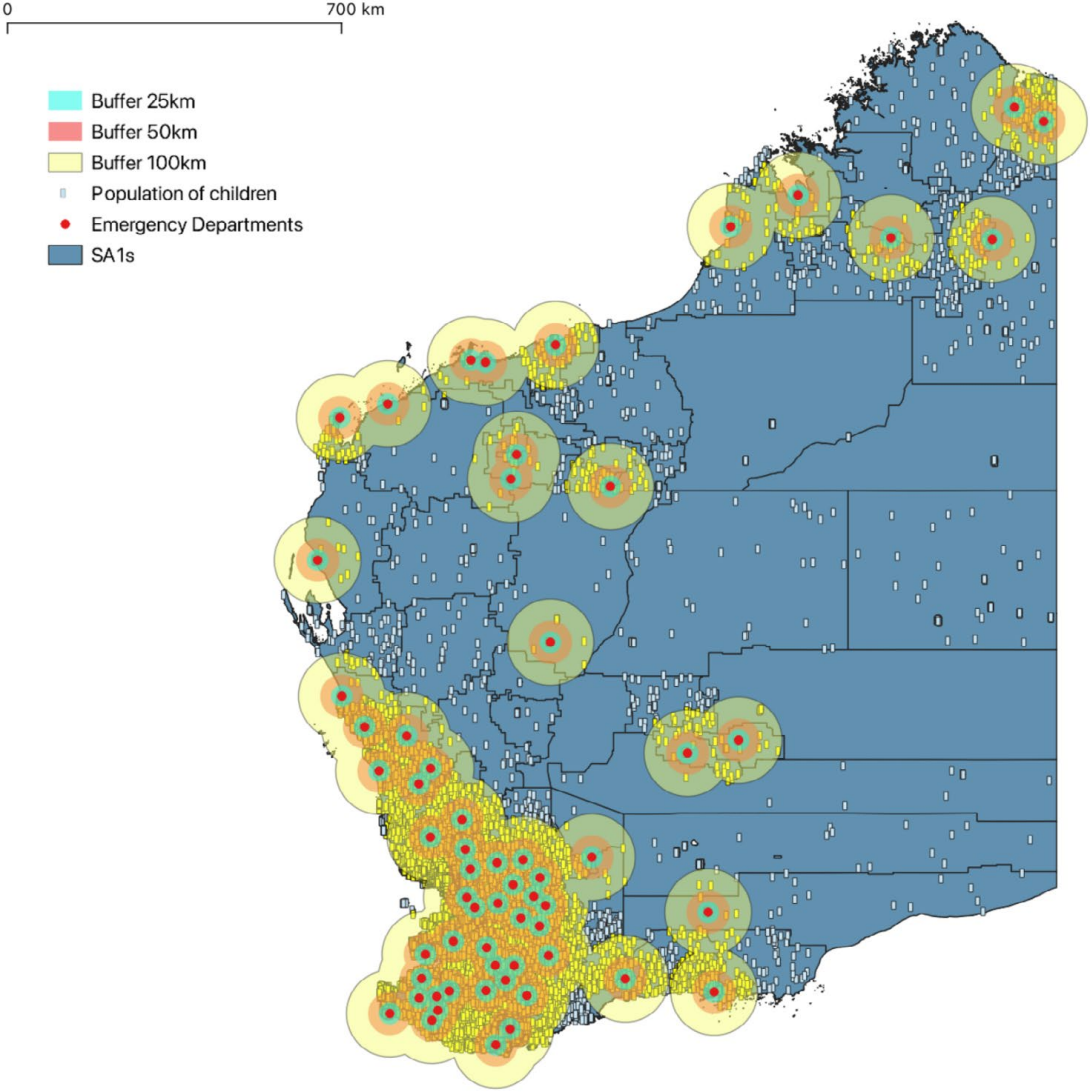
Spatial coverage of emergency departments (red circles) in WA; 25‐ (blue), 50‐ (red) and 100‐km (yellow) buffers around facilities.

Across the three distance buffers, the proportion of children living within reach of an ED increased as the catchment radius expanded, whilst patterns of per capita facility availability varied across MMM5–MMM7 (Figure 3). Within the 25 km buffer, 545 SA1s were included, representing 69.0% (*n* = 36,656) of the study population. ED‐to‐population ratios at this distance indicated substantially greater availability in MMM5 (1:472) and MMM7 (1:459) compared with MMM6 (1:946). Expanding the catchment to 50 km increased the proportion of children living within 50 km of an ED to 81.7% (*n* = 43,385). ED‐to‐child‐population ratios remained lowest in MMM6 (1:1005), whilst MMM5 and MMM7 showed comparatively higher ED availability relative to the child population (1:673 and 1:487, respectively). At 100 km, 92.2% of the study population (*n* = 48,960) lived within 100 km of an ED. The same pattern persisted, with MMM7 showing the highest ED availability relative to the child population (1:556), followed by MMM5 (1:814) and MMM6 (1:995).

## 4 Discussion

This study provides the first paediatric‐focused GIS assessment of spatial accessibility to public, hospital‐based EDs across RR WA. Using graduated distance thresholds, we quantified the proportion of children residing within a defined distance of an ED and examined how accessibility varies by remoteness and area‐level socioeconomic disadvantage. Overall, geographic coverage increased as distance thresholds expanded, but shorter thresholds left a meaningful proportion of children outside local catchments, highlighting persistent geographic inequities in emergency access.

Although the distribution of EDs broadly tracked population patterns across MMM categories, with MMM7 consistently showing the most favourable ED‐to‐child and bed‐to‐child ratios, these ratios do not capture functional capabilities (e.g., staffing mix, on‐site diagnostics and after‐hours coverage) or reliance on retrieval and referral pathways for definitive care. Accordingly, the ratios should not be interpreted as evidence of better emergency access; rather, they highlight that simple resource‐per‐population ratios can diverge from real‐world accessibility in highly remote settings with small populations.

In fact, a significant functional limitation of Australian rural EDs is their structural inability to consistently meet the full complement of resources mandated for accredited EDs, specifically, a lack of consistent 24‐h medical coverage [6]. These limitations are well documented and involve workforce shortages that constrain the capacity of rural EDs to provide after‐hours, urgent and/or emergency care [31], with medical practitioner shortages becoming more pronounced with increasing remoteness [6]. In 2022, there were 205 full‐time equivalents (FTEs) of medical practitioners per 100,000 people in very remote areas, compared with 427 FTEs per 100,000 in major cities [32]. Access to on‐site diagnostic and critical care support is also limited [7]. For example, radiology and pathology services may be absent altogether or restricted to standard business hours [33] and overall resourcing varies by remoteness and community size [7].

Importantly, these constraints affect paediatric readiness, defined as the staffing, equipment and medication availability, protocols, training and quality systems required to deliver safe, timely emergency care to children [34]. In rural contexts, reduced paediatric‐ready capability means that, even when an ED is geographically accessible, it may lack the capacity to definitively manage complex paediatric emergencies [7]. Accordingly, patients presenting to rural EDs are stabilised locally and then transferred to larger regional or metropolitan centres for further investigation and definitive management [35].

Harwood et al. highlight a key paediatric readiness issue in RR settings. Children with acute abdominal pain or suspected appendicitis are frequently retrieved from RR locations to inner‐regional or metropolitan hospitals, in a context where diagnostic testing is intermittent or unavailable. They argue that improving local paediatric capability could support earlier, more accurate assessment and potentially reduce avoidable retrievals [36].

EDs and bed availability were concentrated in IRSD 1 and 2, suggesting an important positive aspect of service planning in WA, where resource planning prioritises the mitigation of the Inverse Care Law by strategically placing services closer to vulnerable populations to promote equitable accessibility. This pattern is consistent with national utilisation statistics indicating a socioeconomic gradient in ED attendance. In 2022–2023, ED utilisation was higher in disadvantaged areas, with IRSD 1 and 2 accounting for nearly half of all ED attendances (24% and 22%, respectively) [8]. Similarly, Bull et al. reported higher early‐life ED presentation rates amongst children (≤ 5 years) from disadvantaged families [37].

### 4.1 Policy Implications

The spatial inequities in paediatric emergency care access identified require multi‐level policy intervention to improve accessibility for rural children. Policy frameworks should prioritise integrated service delivery models linking rural hospitals with tertiary centres through formal clinical networks and standardised care protocols to support timely consultation, escalation and transfer. Emergency tele‐health services must be scaled to provide real‐time specialist consultation and improve point of care decision making [38]. Evidence‐based resource allocation should target areas with greatest accessibility deficits through enhanced retrieval coordination infrastructure (e.g., Royal Flying Doctor Service), standardised transport protocols and community‐based emergency preparedness programmes that build local stabilisation capacity [39].

### 4.2 Strengths and Limitations

This study contributes to the evidence on emergency service accessibility for children in RR regions. A key strength is the application of geospatial mapping to visualise and quantify regional disparities in emergency‐care availability, generating policy‐relevant insights for service planning. However, this study has several limitations that should be acknowledged. First, a small proportion of SA1s had missing IRSD classifications, introducing minor uncertainty into socioeconomic comparisons. Second, the analysis used total hospital bed counts rather than paediatric‐specific ED beds or functional capability measures, thereby limiting conclusions about actual ED capacity for children. Third, accessibility was estimated using fixed‐radius straight‐line buffers instead of road‐network travel time, which may overestimate real‐world access. Finally, the study relied on static population data and did not capture seasonal or temporary population changes, including tourism and fly‐in‐fly‐out workforce movements, which may influence local emergency demand.

## 5 Conclusion

This spatial analysis demonstrates clear geographic and socioeconomic inequities in emergency‐care provision across RR WA. However, these measures do not fully capture the lived accessibility experienced by RR children. Future analyses incorporating network‐based travel impedance will offer a more realistic and policy‐relevant assessment of emergency‐care access.

## Funding

The authors have nothing to report.

## Conflicts of Interest

The authors declare no conflicts of interest.

## Supporting information


**Table S1:** Characteristics and service capability of included rural and remote emergency departments in Western Australia.

## Data Availability

The data that support the findings of this study are available from the corresponding author upon reasonable request.
