# Supplementary material for: Distribution and Accessibility of Hospital‐Based Emergency Departments for Paediatric Patients in Rural and Remote Western Australia: A Geographic Analysis
Source: Emerg Med Australas. 2026 Jun 14;38(3):e70302. doi: 10.1111/1742-6723.70302 (PMC13265136; doi:10.1111/1742-6723.70302)
Supplement: Supplementary file 1 — Table S1: Characteristics and service capability of included rural and remote emergency departments in Western Australia. [file EMM-38-0-s001.docx]

**Supplementary Table 1: Characteristics and service capability of included rural and remote emergency departments in Western Australia.**

| **Country regions** | **Facility name** | **Hospital services** | **Clinical services framework (CSF)-Paediatric services -ED** | **Diagnostics available** | **Paediatric capability** | **Emergency presentations** | **Total hospital bed Numbers** |
| --- | --- | --- | --- | --- | --- | --- | --- |
| **Kimberley** | Broome Regional Hospital | Regional hospital | Level 4 | Medical imaging & Pathology services | Paediatric unit | 25034 | 69 |
|  | Derby District Hospital | District hospital | Level 3 | Medical imaging & Pathology services | No dedicated paediatric unit | 13191 | 40 |
|  | Fitzroy Crossing Hospital | Small hospital | Level 2 | Visiting medical imaging | No dedicated paediatric unit | 7823 | 12 |
|  | Halls Creek hospital | Small hospital | Level 2 | Limited medical imaging & Pathology services | No dedicated paediatric unit | 6794 | 8 |
|  | Kununurra District Hospital | District hospital | Level 3 | Medical imaging & Pathology services | Visiting paediatrics | 13288 | 34 |
|  | Wyndham hospital | Small hospital | Level 1 | Not specified | No dedicated paediatric unit | 3614 | 0 |
| **Pilbara** | Newman Hospital | District hospital | Level 3 | Medical imaging & Pathology services | No dedicated paediatric unit | 8159 | 10 |
|  | Karratha Hospital | District hospital | Level 3 | Medical imaging & Pathology services | Visiting paediatrics | 23131 | 50 |
|  | Hedland Health Campus | Regional hospital | Level 4 | Medical imaging & Pathology services | Paediatric unit | 28069 | 71 |
|  | Onslow Hospital | Small hospital | Level 1 | Digital imaging department | Visiting paediatrics | 1595 | 6 |
|  | Paraburdoo Hospital | Small hospital | Level 1 | Not specified | No dedicated paediatric unit | 948 | 4 |
|  | Roebourne District Hospital | Small hospital | Level 1 | Not specified | No dedicated paediatric unit | 3471 | 10 |
|  | Tom Price District Hospital | Small hospital | Level 1 | Not specified | Visiting paediatrics | 3329 | 8 |
| **Midwest** | Carnarvon Hospital | District hospital | Level 2 | Medical imaging & Pathology services | No dedicated paediatric unit | 8991 | 27 |
|  | Dongara Public hospitals | Small hospital | Level 1 | Medical imaging & Pathology services | No dedicated paediatric unit | 3045 | 3 |
|  | Exmouth Hospital | Small hospital | Level 2 | Medical imaging & Pathology services | No dedicated paediatric unit | 7353 | 9 |
|  | Kalbarri Hospital | Small hospital | Level 1 | Not specified | No dedicated paediatric unit | 3086 | 4 |
|  | Meekatharra Hospital | Small hospital | Level 2 | Not specified | No dedicated paediatric unit | 2267 | 11 |
|  | Morawa Perenjori Hospital | Small hospital | Level 1 | Not specified | No dedicated paediatric unit | 1045 | 5 |
|  | Mullewa Hospital | Small hospital | Level 1 | Not specified | No dedicated paediatric unit | 901 | 0 |
|  | North Midlands Public Hospital | Small hospital | Level 1 | Not specified | No dedicated paediatric unit | 642 | 5 |
|  | Northampton Hospital | Small hospital | Level 1 | Not specified | No dedicated paediatric unit | 943 | 6 |
| **Goldfields** | Esperance Hospital | Regional hospital | Level 3 | Medical imaging & Pathology services | Visiting paediatrics | 11869 | 30 |
|  | Laverton Hospital | Small hospital | Level 1 | Limited medical imaging | Visiting paediatrics | 1604 | 8 |
|  | Leonora Hospital | Small hospital | Level 1 | Limited medical imaging | Visiting paediatrics | 2075 | 8 |
|  | Norseman District Hospital | District hospital | Level 1 | Not specified | No dedicated paediatric unit | 1732 | 8 |
| **Wheatbelt** | Narrogin Regional Hospital | Regional hospital | Level 2 | Medical imaging & Pathology services | No dedicated paediatric unit | 888 | 36 |
|  | Beverley Hospital | Small hospital | Level 2 | Not specified | Child health clinics | Not reported | 7 |
|  | Boddington Hospital | Small hospital | Level 2 | Medical imaging & Pathology services | Child health clinics | Not reported | 8 |
|  | Bruce Rock Memorial Hospital | Small hospital | Level 2 | Not specified | Child health clinics | Not reported | 5 |
|  | Corrigin Hospital | Small hospital | Level 2 | Not specified | Child health clinics | Not reported | 4 |
|  | Dalwallinu Hospital | Small hospital | Level 2 | Medical imaging & Pathology services | Child health clinics | Not reported | 8 |
|  | Dumbleyung Memorial Hospital | Small hospital | Level 1 | Not specified | Child health clinics | Not reported | 4 |
|  | Goomalling Hospital | Small hospital | Level 2 | Not specified | No dedicated paediatric unit | Not reported | 4 |
|  | Kellerberrin Memorial Hospital | Small hospital | Level 1 | Not specified | No dedicated paediatric unit | Not reported | 4 |
|  | Kondinin Hospital | Small hospital | Level 2 | Medical imaging & Pathology services | No dedicated paediatric unit | Not reported | 4 |
|  | Kununoppin Hospital | Small hospital | Level 1 | Medical imaging | Visiting general physician | Not reported | 6 |
|  | Lake Grace Hospital | Small hospital | Level 2 | Not specified | Child health clinics | Not reported | 3 |
|  | Merredin Hospital | District hospital | Level 2 | Medical imaging & Pathology services | No dedicated paediatric unit | Not reported | 16 |
|  | Moora Hospital | District hospital | Level 2 | Medical imaging | No dedicated paediatric unit | Not reported | 12 |
|  | Narembeen Memorial Hospital | Small hospital | Level 1 | Not specified | Child health clinics | Not reported | 3 |
|  | Quairading Hospital | Small hospital | Level 2 | Not specified | Child health clinics | Not reported | 6 |
|  | Southern Cross Health | Small hospital | Level 2 | Not specified | Child health clinics | Not reported | 7 |
|  | Wagin Hospital | Small hospital | Level 2 | Not specified | Child health clinics | Not reported | 8 |
|  | Wongan Hills Hospital | Small hospital | Level 2 | Not specified | Child health clinics | Not reported | 7 |
|  | Wyalkatchem-Koorda Hospital | Small hospital | Level 2 | Not specified | No dedicated paediatric unit | Not reported | 3 |
|  | York Hospital | Small hospital | Level 2 | Not specified | No dedicated paediatric unit | Not reported | 7 |
| **Southwest** | Augusta Hospital | Small hospital | Level 2 | Not specified | No dedicated paediatric unit | 1542 | 9 |
|  | Boyup Brook Memorial Hospital | Small hospital | Level 1 | Not specified | No dedicated paediatric unit | 914 | 5 |
|  | Bridgetown Hospital | Small hospital | Level 3 | Not specified | No dedicated paediatric unit | 3993 | 25 |
|  | Donnybrook Hospital | Small hospital | Level 2 | Not specified | No dedicated paediatric unit | 1363 | 14 |
|  | Harvey Hospital | Small hospital | Level 2 | Not specified | No dedicated paediatric unit | 3057 | 22 |
|  | Nannup Hospital | Small hospital | Level 2 | Not specified | No dedicated paediatric unit | 571 | 2 |
|  | Pemberton Hospital | Small hospital | Level 2 | Not specified | No dedicated paediatric unit | 947 | 9 |
|  | Manjimup Hospital (Warren Health Service) | District hospital | Level 3 | Pathology services | No dedicated paediatric unit | 5890 | 18 |
| **Great Southern** | Katanning Health Service | District hospital | Level 3 | Medical imaging & Pathology services | No dedicated paediatric unit | 5309 | 10 |
|  | Denmark Hospital | Small hospital | Level 2 | Not specified | No dedicated paediatric unit | 4082 | 7 |
|  | Gnowangerup Hospital | Small hospital | Level 2 | Not specified | No dedicated paediatric unit | 666 | 4 |
|  | Kojonup Hospital | Small hospital | Level 2 | Not specified | No dedicated paediatric unit | 901 | 6 |
|  | Plantagenet Cranbrook Hospital | Small hospital | Level 2 | Not specified | No dedicated paediatric unit | 2944 | 11 |
|  | Ravensthorpe Health service | Small hospital | Level 2 | Limited medical imaging & Pathology services | No dedicated paediatric unit | 1419 | 4 |
